# Antibodies and Inflammation: Fecal Biomarkers of Gut Health in Domestic Ruminants

**DOI:** 10.1002/jez.2896

**Published:** 2025-01-22

**Authors:** A. M. Burton, K. J. Else, J. Irving, I. Mair, S. Shultz

**Affiliations:** ^1^ Department of Earth and Environmental Science, School of Natural Sciences, Faculty of Science and Engineering The University of Manchester Manchester UK; ^2^ Lydia Becker Institute of Immunology and Inflammation, School of Biological Sciences, Faculty of Biology, Medicine and Health The University of Manchester Manchester UK; ^3^ Division of Immunology, Immunity to Infection and Respiratory Medicine, School of Biological Sciences, Faculty of Biology, Medicine and Health The University of Manchester Manchester UK; ^4^ Institute of Ecology and Evolution, Institute of Immunology and Infection Research, School of Biological Sciences The University of Edinburgh Edinburgh UK

**Keywords:** antibodies, ecoimmunology, inflammation, lactoferrin, non‐invasive biomarkers

## Abstract

Gastrointestinal infections present major challenges to ruminant livestock systems, and gut health is a key constraint on fitness, welfare, and productivity. Fecal biomarkers present opportunities to monitor animal health without using invasive methods, and with greater resolution compared to observational metrics. Here we developed enzyme‐linked immunosorbent assays for three potential fecal biomarkers of gut health in domestic ruminants: two immunological (total immunoglobulin [Ig]A and total IgG) and one inflammatory (lactoferrin). We analytically validated the assays, then evaluated whether they could be used as a biomarker of clinically diagnosed gastrointestinal pathologies in cattle (*Bos taurus*), and finally compared them with helminth fecal egg counts in sheep (*Ovis aries*). The analytes were detected above the lower limits of detection in cattle, sheep, and goats. Fecal IgA and lactoferrin were higher in cattle with infectious pathologies (strongyles, coccidiosis and symptomatic Johne's disease) compared to healthy controls. Lactoferrin was additionally higher in animals with infectious pathologies compared to noninfectious pathologies, and to asymptomatic Johne's cases. No significant relationships were found with sheep fecal egg counts. These initial findings suggest that fecal IgA and lactoferrin may be useful biomarkers of poor gastrointestinal health in cattle, and that fecal lactoferrin is specific to active inflammation caused by infectious agents. These could be incorporated into the growing suite of noninvasive ecoimmunological tools and used to understand ruminant gut health in a range of species. Applications include improving treatment regimens for gastrointestinal infections, and understanding wildlife physiological responses to infectious challenges.

## Summary

- Fecal biomarkers can distinguish gastrointestinal pathologies in cattle.
- Infectious pathology cases had elevated IgA and lactoferrin compared to controls, and elevated lactoferrin compared to non‐infectious pathologies and asymptomatic Johne's.

## 1 Introduction

Gut health is a key constraint to overall animal health, welfare, and productivity in ruminant livestock systems. Infectious gastrointestinal diseases are common, presenting widespread economic and animal welfare challenges. Parasitic helminths, for example, are one of the most common gastrointestinal infections globally, resulting in reduced body condition, growth and reproductive rates (Fox 1997). In Europe alone, the cost of parasitic helminth infections in ruminant livestock was an estimated €1.8 billion annually (Charlier et al. 2020), therefore methods to monitor and mitigate their impacts could be financially valuable. Johne's disease (or paratuberculosis) is another common gastrointestinal disease in domestic ruminants, a chronic enteritis caused by *Mycobacterium avium* subspecies *paratuberculosis* (MAP). Clinical and advanced stages of the disease include weight loss, diarrhea, drop in milk production, and eventually death due to wasting and dehydration (Matthews, Cotter, and O'Mahony 2021). Animals can, however, also be infected in silent and subclinical stages of the disease, resulting in the “iceberg phenomenon”, where the apparent number of detectible cases of MAP in any given population is an underestimation of the true prevalence (Magombedze, Ngonghala, and Lanzas 2013; Whitlock and Buergelt 1996). Other common gastrointestinal diseases include coccidiosis (caused by host‐specific *Eimeria spp*. protozoan parasites [Bangoura and Bardsley 2020]), winter dysentery (thought to be caused by bovine coronavirus [Alenius et al. 1991]), hemorrhagic bowel syndrome (which has unknown etiology, but proposed causative agents include *Clostridium perfringens* type A and *Aspergillus fumigatus* [De Jonge et al. 2023]), giardiasis, and cryptosporidiosis (caused by the protozoans *Giardia spp*. and *Cryptosporidium spp*. respectively [O'handley and Olson 2006]). Infectious gastrointestinal diseases therefore represent a substantial challenge to ruminant livestock production systems, and noninvasive biomarkers of pathologies could provide new ways to detect and monitor infection and the progression of disease (Celi et al. 2019; Rathod and Dhok 2024).

In addition to infectious disease, there are other factors regulating ruminant gastrointestinal functionality. The gut mucosa protects the mammalian body against infections, regulates immune function, and controls the digestion and absorption of nutrients (Celi et al. 2017). In ruminants, the gastrointestinal epithelia is split into two distinct types; the stratified squamous epithelia of the reticulum, rumen, and omasum, and the mucosal columnar epithelium of the abomasum, small intestine, cecum, and large intestine (Steele et al. 2016). The mucus layers are composed predominantly of mucin networks, and form the barrier between the microbiota and the host (Kim and Ho 2010). Maintaining mucosal barrier integrity is a critical process; it is continually challenged with parasites, and contact with digestive enzymes, foodstuffs, and the micro‐ and mycobiota (Celi et al. 2017), and yet must also be selectively permeable to nutrients (Bischoff 2011; Bischoff et al. 2014). Developing new methods to measure gastrointestinal functionality is a key aim for improving animal welfare and veterinary medicine (Celi et al. 2019; Rathod and Dhok 2024).

Invasive sampling of tissue or serum is commonly used to measure antibodies and other biomarkers, to indicate the presence or burden of pathogens in the host. This can be costly and stressful for the animal, particularly for wildlife where capture is required (Breed et al. 2019). There are also methodological challenges in interpreting results, as antibody concentrations can vary between tissue, serum, and mucosal samples (e.g., due to tissue‐specific immunity [Wu et al. 2019]). Alternative noninvasive methods to measure animal health (including body condition scores, fecal consistency scoring, and behavioral indicators) can be crude and subjective, and are not consistently linked with parasite burden or pathology (Sánchez et al. 2018). As such, there has been an increased effort to develop fecal markers of health, to objectively and precisely measure this variation in a wide range of species, and in ecological and veterinary contexts (Rathod and Dhok 2024). Some of these markers include lactate and succinate (Sato and Koiwa 2008), sialic acid (Huang et al. 2015), and intestinal alkaline phosphatase (Lallès 2010). In addition, markers of the immune state (i.e., the overall immune phenotype of an animal at any given timepoint) are increasingly being explored, as will be introduced in more detail in the following paragraphs.

Defense against pathogens and parasites, and the development of cell memory, requires an adaptive immune response. Immunoglobulins (Igs) are antigen‐specific glycoproteins secreted from (or present on the surface of) B cells, as part of the adaptive immune response. The specific combination of Ig isotopes (IgA, IgD, IgE, IgG, IgM) and their sub‐classes will depend on the type of infectious challenge (Bonilla and Oettgen 2010), representing a diverse range of antibody‐mediated effector functions. Two isotopes were investigated in this study: IgA and IgG. IgA is the most prevalent immunoglobulin at mucosal membranes (Brandtzaeg et al. 1999). It is typically found in high concentrations in excretory products such as feces, due to its ability to form the dimeric molecule secretory IgA (sIgA), which is more resistant to degradation, and which is actively secreted across mucosal linings (Kaetzel, Mestecky, and Johansen 2017). sIgA provides nonspecific protection through immune exclusion; the process of microorganisms being trapped and clumping together (agglutination and entrapment), before being cleared by peristaltic bowel movements (Li, Jin, and Chen 2020; Mantis, Rol, and Corthésy 2011). sIgA also has more specific immune functions such as blocking pathogens interacting with the epithelium, and reducing bacterial viability or pathogenicity (e.g., Apter et al. 1993; Forbes, Eschmann, and Mantis 2008), and it plays a crucial role in regulating the gastrointestinal microbiota (Rollenske et al. 2021). Finally, sIgA interacts with neutrophils, eosinophils, monocytes, dendritic cells, and Kupffer cells through surface receptors, mediating immune processes including antigen presentation, cytokine release, phagocytosis and antibody‐dependent cellular cytotoxicity (ADCC) (Li, Jin, and Chen 2020; Monteiro and van de Winkel 2003). IgG is the predominant class of circulating serum immunoglobulins (Nezlin 1998), in addition to being the most abundant in bovine colostrum and milk (Geiger 2020). It is highly associated with gastrointestinal helminths (alongside IgE), due to the strong T helper (Th)‐2 immune response to clear the parasites; helminth infections trigger the production of cytokines including interleukin‐4, which is associated with class switching in B cells to produce IgG and IgE (Logan, Chetty, and Horsnell 2014). In livestock, IgG is involved in antigen recognition of economically important parasites such as *Ostertagia ostertagi* (Canals and Gasbarre 1990) and *Fasciola gigantica* (Ogunrinade 1983). Mucosal IgG is also important in regulating commensal gut microbiota (Chen et al. 2020), and dysregulated IgG responses can drive inflammation, through eliciting Th‐17 cell population expansion (Castro‐Dopico et al. 2019). IgE was not investigated in this study, although serum IgE is thought to be critical in the immune response to helminth parasites in humans and animals (Cooper et al. 2008; Gershwin 2009; Shaw, Pfeffer, and Bischof 2009). IgE binds to innate immune cells (e.g., mast cells and basophils), sensitizing them and leading to degranulation (Marshall et al. 2018). It is, therefore, typically found in very low or negligible concentrations in feces, particularly compared to sIgA. Fecal anti‐*Teladorsagiacircumcincta* IgE has been undetectable in Soay sheep (Watt et al. 2016) and cattle (Cooke et al. 2019), despite positive results from plasma in these animals.

In general, an upregulation in antibody concentrations is thought to indicate an increasing response to immune challenges, however antibody concentrations will fluctuate during the course of an infection and depending on a multitude of host‐ and pathogen‐specific contexts. Antibody production can begin a few days to weeks following infectious challenges, usually built over time, and can remain high following the clearance of infection or can decline over time (Janeway et al. 2001). In human patients with inflammatory bowel disease (IBD), both IgA and IgG have been found to be upregulated compared to healthy controls, and to correlate with disease severity in patients with Crohn's disease (Lin et al. 2018; Liu et al. 2020). In some circumstances, however, antibody levels are not a good indicator of immunity. Some filarial nematodes, for example, can modulate or evade the immune system, reducing the efficacy of the adaptive immune response (MacDonald, Araujo, and Pearce 2002; Taylor et al. 2001). Antibody concentrations should, therefore, be considered in disease‐specific contexts, and a suite of biomarkers may be needed to unravel the impacts of infections on gut health.

Another key part of mucosal defense is inflammation, triggered by both the innate and adaptive immune responses (Cronkite and Strutt 2018). While controlled inflammation is an adaptive process that protects the mucosa from damage, if it is not resolved effectively it can develop into chronic inflammatory disorders (Lawrence and Gilroy 2007). Fecal biomarkers of gastrointestinal inflammation are increasingly being used within studies of animal health, including lipocalin‐2 (Chassaing et al. 2012), calprotectin (Bogere, Choi, and Heo 2019; Heilmann et al. 2018), and lactoferrin. Lactoferrin is an iron‐binding neutrophilic glycoprotein associated with gastrointestinal inflammation (Kell, Heyden, and Pretorius 2020). It is bacteriostatic and bactericidal (Rainard 1986; Yamauchi et al. 1993), can provide defense against a broad range of fungi, protozoa, and viruses (Orsi 2004), and has been shown to be parasiticidal, both in vitro and in animal models (León‐Sicairos et al. 2017). It is a desirable target as a fecal biomarker as it is resistant to proteolysis and to freeze‐thaw cycles, and can remain stable for up to 7 days at room temperature or when refrigerated (Abraham 2018; Guerrant et al. 1992). Fecal lactoferrin is commonly used as a specific and sensitive marker of intestinal inflammation in humans, and it is positively associated with disease severity in inflammatory bowel diseases such as Crohn's disease (Klimczak et al. 2015; Mosli et al. 2015; Sidhu et al. 2010). Bovine lactoferrin is used commercially within human and veterinary medical industries, due to its many therapeutic roles (Abdelnour et al. 2023; Ashraf et al. 2023), therefore bovine‐specific antibodies and standards are more widely available compared to other potential inflammatory biomarkers.

Fecal detection of ruminant antibodies has been shown in domestic cattle (*Bos taurus*) (Cooke et al. 2019) and wild Soay sheep (*Ovis aries*) (Hayward et al. 2019; Watt et al. 2016), both in response to natural infections of gastrointestinal helminths. Fecal lactoferrin has also been successfully detected in cattle, comparing fecal, serum, and milk concentrations (Cooke et al. 2020). The association of these markers with clinically diagnosed infectious gastrointestinal pathologies, however, has not yet been tested. Here, we compare the biological validity of three potential fecal biomarkers of poor gut health in domestic ruminants: total IgA, total IgG, and lactoferrin. These were chosen to give an overview of gastrointestinal mucosal immunity, response to extracellular infections, and inflammation. In comparing individuals with clinical diagnoses of infectious and noninfectious gut pathologies to healthy controls, we aim to provide evidence that fecal biomarkers can be used to effectively monitor gut health in ruminants. We predict that IgA will be elevated nonspecifically in response to infectious agents, IgG will be elevated with gastrointestinal helminth burden, and that lactoferrin will be elevated in animals with inflammatory pathologies, in particular with cases of active inflammation.

## 2 Methods

### 2.1 Ethical Statement

This project was conducted with approval for Category D research from the University of Manchester Research Governance, Ethics and Integrity Committee (reference number: D064).

### 2.2 Samples

Fecal samples were provided by Willows Farm Vets, Northwich, UK. Samples were collected by vets during routine visits and were stored frozen at −20°C before transport to the University of Manchester, UK, for extraction and analysis. Samples were collected from cattle (*Bos taurus*) (*n* = 16), sheep (*Ovis aries*) (*n* = 10), and goats (*Capra aegagrus hircus*) (*n* = 3). Cattle samples were grouped based on the diagnoses and symptoms listed by the collecting vets: control samples (*n* = 5; no evidence of gastric pathology), noninfectious pathology (*n* = 5; displaced abomasum, toxin ingestion), infectious pathology (*n* = 3; symptomatic Johne's disease, strongyle infections, coccidiosis), and asymptomatic Johne's (*n* = 3, antibody positive for Johne's disease without any clinical symptoms). Sheep and goat samples were collected for routine faecal egg counts (FECs), conducted by Willows Farm Vets, and no other symptoms were reported. Routine FECs were not performed on cattle samples unless strongyle infections were suspected (*n* = 1).

### 2.3 Extraction

The fecal sample extraction protocol was modified from Tombak et al. (2020). A protease inhibitor cocktail (Generon, M5293‐1) was diluted 1:100 in 1× phosphate‐buffered saline (PBS) to give the 1× PBS‐Protease inhibitor (PBS‐PI) extraction buffer. Fecal samples were thawed and homogenized, and 0.5 g added to 500 μL PBS‐PI (1:1 weight: volume). Where fecal matter was drier (goats and some sheep samples), 1:1.5 weight: volume was used. Samples were left to soften for 5 min, then a clean toothpick was used to break up feces and ensure complete mixing with the extraction buffer. These were left to incubate for 20 min at room temperature, then centrifuged for 5 min at 15,000*g*, and the supernatant was removed. The fecal extract was stored at −20°C until assays were run.

### 2.4 Assays

Fecal antibodies were measured using sandwich enzyme‐linked immunosorbent assays (ELISAs). Antibodies and standards used for each assay, and their working concentrations, are listed in Table 1. Coating antibodies were diluted to the working concentration in 0.05 M carbonate‐bicarbonate buffer (Sigma‐Aldrich, C3041‐50CAP), and 96‐well half area ELISA microplates (Grenier Bio‐One 675061) were coated overnight at 4°C. Coated plates were stored at 4°C for a maximum of 3 days. All samples were diluted to the working dilution using 1× phosphate buffered saline containing 0.1% Tween‐20 (PBS‐T). Standards were diluted to the starting concentration in PBS‐T, then serially diluted to generate a standard curve. Coated plates were washed 5 times with PBS‐T and tapped dry. Samples, standards, and controls were run in duplicate, using 25 μL per well. PBS‐T was used as a negative control on all plates. Plates were then incubated for 1 h in the dark, at 37°C (IgA and IgG) or room temperature (lactoferrin), then washed five times with PBS‐T, and tapped dry. 25 μL HRP‐conjugated detection antibody was added to each well (Table 1), followed by another incubation for 1 h at 37°C (IgA and IgG) or room temperature (lactoferrin), and another round of washing. Finally, 35 μL 3, 3′, 5, 5′‐tetramethylbenzidine (TMB) was added to each well (BioLegend UK Ltd., 421101; equal parts A and B were combined in an opaque tube immediately before use), and they were incubated in the dark at room temperature to allow the color to develop. Reactions were stopped with 35 μL 1 M H_2_SO_4_. Final incubation times were 3 min for IgA, 4 min for IgG, 10 min for Lactoferrin. Optical densities (ODs) were read at 450 nm, immediately after the reaction was stopped, and again 10 min later. Uniformity of background readings was checked using 620 nm.

**Table 1:** Antibodies and standards used in each assay, with working concentrations.

Assay	Coating antibody	Conc. (μg/mL)	Detection antibody	Conc. (μg/mL)	Standard	Starting conc. (μg/mL)
IgA	Rabbit anti‐Bovine IgA Heavy Chain Antibody Affinity Purified, 1 mg, Cambridge Bioscience Ltd. #A10‐108A	1	Rabbit anti‐Bovine IgA Heavy Chain Antibody HRP Conjugated, Cambridge Bioscience Ltd. #A10‐108P	0.125	Bovine IgA (nonimmune, serum) purified, 2B Scientific #20001‐3‐1‐AD	2
IgG	Rabbit anti‐Bovine IgG Heavy and Light Chain Antibody Affinity Purified 1 mg, Cambridge Bioscience Ltd. #A10‐102A	2	Rabbit anti‐Bovine IgG Heavy and Light Chain Antibody HRP Conjugated 1 mg, Cambridge Bioscience Limited #A10‐102P	0.0625	Purified Bovine IgG, Bethyl Laboratories Inc #P10‐115	2
Lactoferrin	Goat anti‐Bovine Lactoferrin Antibody Affinity Purified, 1 mg, Cambridge bioscience #A10‐126A	2	Goat anti‐Bovine Lactoferrin Antibody HRP Conjugated 1 mg, Cambridge bioscience #A10‐126P	0.03125	Lactoferrin from bovine colostrum, ≥ 85% (SDS‐PAGE), Sigma‐Aldrich #L4765‐10MG	1

### 2.5 Analytical Validation

Assays were analytically validated using linearity (in cattle and sheep samples) and spike‐recovery tests (in cattle samples). Goat samples were excluded due to low sample sizes. Fecal extracts were combined into a pool and diluted to the starting concentration, and an aliquot was spiked with the corresponding standard. The starting dilution and spiking concentration for each assay were determined by preliminary tests of serially diluted un‐spiked samples, to determine the optimal range for each target in the cattle extracts. Spiking concentrations were 500 ng/mL for IgA, 31.25 ng/mL for IgG, and 62.5 ng/mL for lactoferrin. The spiked and un‐spiked pools were serially diluted five times within the linear range of detection for each assay (IgA and IgG used a 1.5‐fold dilution factor, lactoferrin used twofold), and concentrations compared to calculate linearity (Supporting Information S1: Equation 1) and recovery (Supporting Information S1: Equation 2). Average linearities and recoveries were calculated for each assay. Samples were run in duplicate, and intra‐plate coefficients of variance (CV) > 10% were repeated. The lower limit of detection (LOD; Supporting Information S1: Equation 4) was calculated based on the lower limit of the blanks (LOB; Supporting Information S1: Equation 3), and the lowest standard dilution run for each assay (Armbruster and Pry 2008).

### 2.6 Statistical Analysis

All concentrations were converted to ng/g fecal matter (Supporting Information S1: Equation 5), and all statistical analyses were performed in R (R Core Team 2023). Biomarker concentrations and FEC were log‐10 transformed due to right skewed distributions. Transformed data were inspected visually for normal distributions using histograms and QQ plots and tested using Shapiro–Wilk's method. Cattle group differences were compared using a one‐way, independent measures ANOVA with post‐hoc Tukey's Honest Significant Difference (HSD) tests. Pearson's correlations were used to check for correlations between biomarkers, and linear regressions between concentrations in sheep to FEC. Data visualization used the R packages ggplot2 (Wickham 2016), ggsignif (Ahlmann‐Eltze and Patil 2021) and ggthemes (Arnold 2012), with custom themes (Desiraju 2020).

## 3 Results

### 3.1 Analytical Validity

**Figure 1:**
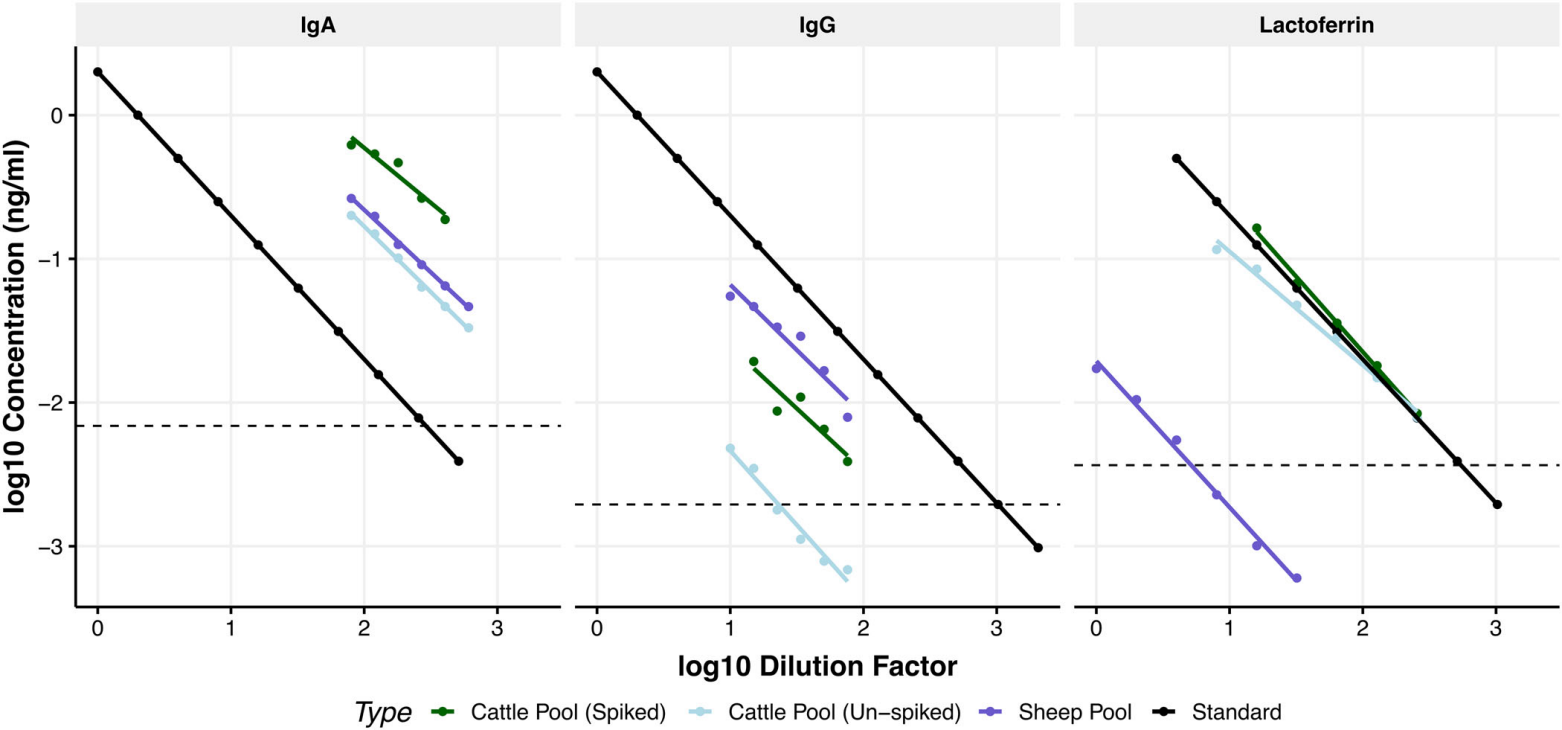
Linearities and spike‐recoveries of bovine IgA, IgG, and Lactoferrin in cattle and sheep fecal samples. Negative correlations between log10 dilution factor and log10 concentration (ng/mL) for the standard curve (black), the serially diluted un‐spiked cattle fecal pool (light blue), the serially diluted spiked fecal cattle pool (green), and the serially diluted sheep pool (purple). Spiking concentrations were 500 ng/mL for IgA, 31.25 ng/mL for IgG, and 62.5 ng/mL for lactoferrin. Dashed horizonal line shows the calculated lower limit of detection, based on the calculated limit of blanks and lowest standard dilution.

Log‐10 transformed biomarker concentrations were not significantly different from normal distributions (IgA: *W* = 0.95, *p* = 0.20, IgG: *W* = 0.97, *p* = 0.69, lactoferrin: *W* = 0.96, *p* = 0.36). The average intra‐plate CV for IgA was 5.99%, for IgG was 3.45%, and for lactoferrin was 2.90%. The average inter‐plate CV for IgA was 24.10%, for IgG was 11.66%, and for lactoferrin was 19.26%. Average linearities for spiked and un‐spiked fecal pools, and average recoveries, were within the normal range for all assays (80%–120%) (Figure 1, Table 2). The LOD was calculated for IgA; 6.88 ng/mL, IgG; 1.95 ng/mL, and lactoferrin; 3.67 ng/mL. Although some serially diluted samples in the linearity test fell below this calculated LOD (Figure 1), they were still detected above the LOB, and individual samples included in the subsequent analysis were not diluted past the LOD. All three biomarkers were successfully measured in cow, sheep, and goat samples, resulting in concentrations above the LOD (Supporting Information S1: Figure S1).

**Table 2:** Linearity and spike‐recovery tests for each assay in cattle and sheep.

Assay	Average linearity (%)			*R* ^2^			Average recovery (%)
	Cattle (un‐spiked)	Cattle (spiked)	Sheep	Cattle (un‐spiked)	Cattle (spiked)	Sheep	
IgA	104.83	112.93	106.22	1.00	0.96	1.00	128.58
IgG	96.29	108.61	104.42	0.97	0.86	0.92	80.47
Lactoferrin	110.53	95.56	103.42	1.00	1.00	1.00	117.80

### 3.2 Biomarker Correlations

**Figure 2:**
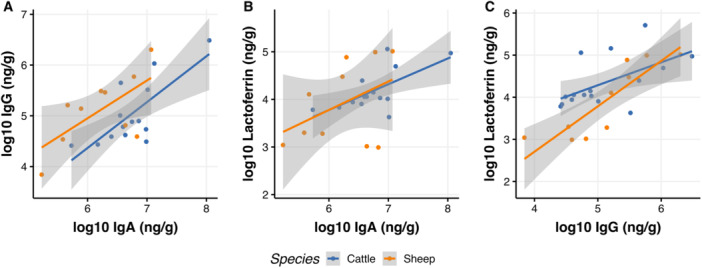
Positive correlations between fecal IgA and IgG (A), IgA and lactoferrin (B), and IgG and lactoferrin (C), in cattle and sheep samples. Linear models showing relationships between concentrations (log10 ng/g) of fecal IgA, IgG, and lactoferrin in cattle (blue) and sheep (orange) samples. The shaded area shows standard error.

Positive correlations were found between all three biomarkers in cattle and sheep fecal samples, with the exception of IgA and lactoferrin in sheep (Figure 2, Table 3).

**Table 3:** Correlations between fecal biomarkers in cattle and sheep samples.

Pair	Cattle					Sheep				
	*t*	*df*	*r*	*p*		t	*df*	*r*	*p*	
IgA–IgG	3.89	12	0.75	0.002	**	2.43	8	0.3	0.04	*
IgA–Lactoferrin	2.971	12	0.65	0.01	*	1.31	8	0.42	0.23	
IgG–Lactoferrin	2.54	14	0.56	0.02	*	4.91	8	0.87	0.001	**

*p < 0.05; **p < 0.01.

### 3.3 Response Gastrointestinal Pathology and Parasites

The three fecal biomarkers were then applied to fecal samples from cattle which had been classified into four pathology groups; healthy controls, noninfectious pathologies, infectious pathologies and asymptomatic Johne's, and from sheep in the context of their fecal eggs counts as a proxy of parasite worm burden.

**Figure 3:**
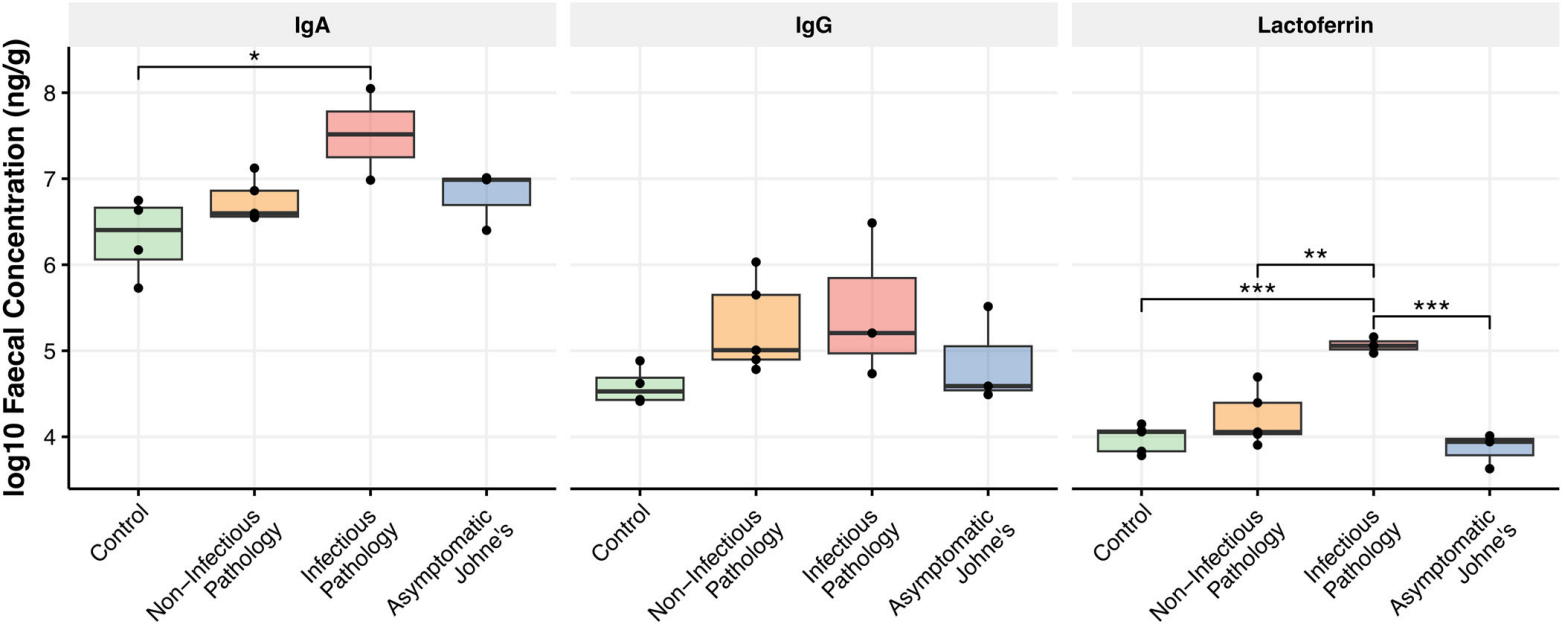
Fecal IgA, IgG, and lactoferrin in cattle with different gastrointestinal pathologies. Concentrations (log10 ng/g) of fecal IgA, IgG, and lactoferrin in cattle with no pathologies (control), noninfectious pathologies, infectious pathologies, and asymptomatic Johne's. Significance bars show *p*‐values from post‐hoc Tukey HSD tests. Lactoferrin was higher in infectious pathologies compared to all other groups. IgA was higher in infectious pathologies compared with controls.

In cattle, the concentration of fecal lactoferrin varied between the four treatment groups (*F*_(3,12)_ = 18.04, *p* < 0.001), and post‐hoc Tukey HSD tests showed higher concentrations in the infectious pathology compared to the controls (difference in means = 1.09, 95% confidence intervals = 0.59 and 1.58, *p* < 0.001), to non‐infectious pathologies (difference in means = 0.85, 95% confidence intervals = 0.35 and 1.43, *p* = 0.001), and to asymptomatic Johne's cases (difference in means = 1.20, 95% confidence intervals = 0.65 and 1.76, *p* < 0.001) (Figure 3). IgA also varied between treatment groups (*F*_(3,10)_ = 3.73, *p* = 0.05), and was higher in infectious pathologies than controls (difference in means = 1.19, 95% confidence intervals = 1.00 and 2.29, *p* = 0.03). A similar pattern was observed for IgG, although this was not statistically significant.

**Figure 4:**
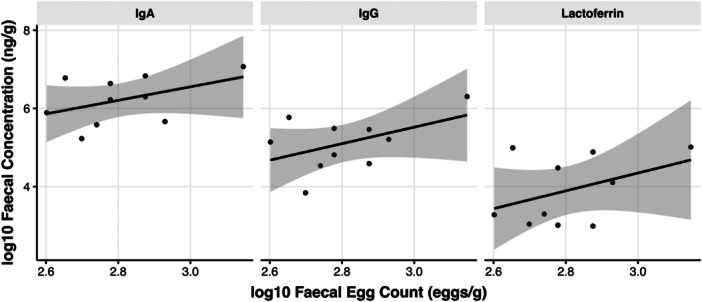
Fecal IgA, IgG, and lactoferrin in sheep showed weak positive associations with fecal egg count. Linear models showing relationships between concentrations (log10 ng/g) of fecal IgA, IgG, and lactoferrin in sheep compared to helminth FEC as a proxy of parasite burden. The shaded area shows standard error. Linear models were not significant for any biomarker.

In sheep, weak positive slopes were seen between FEC and IgA (estimate = 0.11, *t* = 1.40, standard error (SE) = 0.08, *p* = 0.20), IgG (estimate = 0.11, t = 1.52, SE = 0.07, *p* = 0.17), and lactoferrin (estimate = 0.07, t = 1.27, SE = 0.05, *p* = 0.24), although these were not statistically significant (Figure 4). In addition to the small sample size for sheep in this study, the range of FEC values was relatively narrow; 6/10 samples had a moderate infestation (400–600 eggs/g), and no samples had a light infestation (Stubbings et al. 2020). Using a larger sample size, with a wider range of FECs from low to high, could enable future studies to discern more accurately if there is a positive relationship between parasite egg shedding and biomarker concentrations in domestic sheep.

## 4 Discussion

### 4.1 Biomarker Response to Gastrointestinal Infections

In this study we tested the use of fecal immune and inflammatory biomarkers to detect infectious gastrointestinal pathologies in domestic cattle and sheep. We found significant positive correlations between fecal IgA, IgG, and lactoferrin, which is consistent with other literature on fecal antibodies in ruminants (Cooke et al. 2019; Hayward et al. 2019; Watt et al. 2016). Concentrations of fecal lactoferrin were significantly higher in animals with gastrointestinal pathologies caused by infectious agents compared with healthy controls, noninfectious pathologies, and animals that had tested positive for Johne's disease (using the ELISA blood test for antibodies against *Mycobacterium avium* subspecies *paratuberculosis* [MAP]) but that weren't displaying clinical symptoms. These preliminary results suggest that fecal lactoferrin could be a sensitive marker of infection‐induced gastrointestinal inflammation in bovids. This is consistent to work in human medicine, where fecal lactoferrin has been shown to be higher in active cases of Crohn's disease and ulcerative colitis compared to inactive cases and healthy controls (Chen et al. 2012; Klimczak et al. 2015; Sidhu et al. 2010). Moreover, in dogs with canine diarrhea, fecal lactoferrin was higher in infectious diarrhea (caused by parasites, viruses, or bacteria), compared to healthy controls and to non‐infectious, nutritional causes of diarrhea (Maden and Gülersoy 2023). Our preliminary findings in cattle support the idea that fecal lactoferrin could become a sensitive marker, not only of the severity of gastrointestinal inflammation, but also of its' etiology.

Of the three biomarkers, IgA was found in the highest concentrations in the feces, as was expected, as it is the most abundant antibody in mucosal surfaces (Cooke et al. 2019; Lamm 1988; Snoeck, Peters, and Cox 2006). Fecal antibodies in cattle showed a similar pattern to lactoferrin, and IgA was significantly higher in the infectious pathology group than controls. IgA is known to be associated with the immune response against gastrointestinal infections in ruminants, particularly nematodes (Arsenopoulos, Symeonidou, and Papadopoulos 2017; Claerebout and Vercruysse 2000; de la Chevrotière et al. [2012]), however, fecal antibody testing is still a relatively new method in cattle. Cooke et al. (2019) were also able to measure both total and parasite‐specific IgA, IgG, and IgM in fecal samples, but did not find a significant association with parasite burden.

In sheep, although weak positive trends were observed between biomarkers and fecal egg count (FEC), these were not significant correlations. Relationships between fecal antibodies and helminth parasites in sheep are complex in the literature; in wild Soay sheep, parasite‐specific fecal IgA was negatively associated with FEC when controlling for age and sex variation, and total IgG was positively associated (when accounting for the negative anti‐parasite IgA relationship) (Watt et al. 2016). Moreover, parasite‐specific fecal IgG was negatively associated with FEC, and total IgM positively correlated, in the same population (Hayward et al. 2019). These studies in wild sheep utilised larger sample sizes than were available to us in the present study, therefore the absence of statistical significance may not be a true rejection of the hypothesis in these clinical samples. In domestic sheep, genetic resistance to *Haemonchus contortus* is associated with the IgG subclass IgG_1_ (Gill et al. 1994, 1993), whereas resistance to *Teladorsagiacircumcincta* is associated with IgG_2_ and IgA, suggesting different immune effector mechanisms for these two nematode parasites. Total IgG was used in this study due to the inclusion of multiple different infectious agents, therefore future work could investigate antibody sub‐classes (cattle have three IgG sub classes (Rabbani et al. 1997) with distinct functions (Noble et al. 2023), or parasite‐specific antibodies if a single host‐parasite system is of interest.

One current limitation of this toolkit is limited understanding of how confounding variables, particularly co‐infections, impact gastrointestinal health and immune responses. Gastrointestinal helminths have been associated with increased susceptibility to bacterial co‐infections, due to modulation of the immune response. In cattle for example, infection with *Fasciola hepatica* reduces the *Mycobacterium bovis*—specific Th1 response, reducing the sensitivity of common tests for bovine tuberculosis (Flynn et al. 2007). Helminth infections are also thought to drive seasonality in anthrax (*Bacillus anthracis*) in plains zebra (*Equus quagga*), again due to increased resource allocation towards Th‐2 responses, preventing an effective Th‐1 response against anthrax infection (Cizauskas et al. 2014). The relationships between parasitic infection dynamics and fecal immune biomarkers in naturally infected populations may be more complex than predicted by laboratory and experimental studies. Another potentially confounding factor is the integrity of the gastrointestinal epithelial lining, and its impact on gut passage dynamics. Increases in intestinal permeability can be caused by helminths damaging the epithelium, but also by the host, as an active way to transport antibodies and immune cells into the lumen (Bąska and Norbury 2022; McKay, Shute, and Lopes 2017). Other factors such as the gut microbiota, nutrition, and exercise, can also alter intestinal permeability, which may alter the composition of the feces (Dmytriv, Storey, and Lushchak 2024). Changes in fecal biomarker composition may, therefore, reflect the health of the gastrointestinal lining, in addition to specific immune responses to infections.

### 4.2 Applications

With further validations, there are several potential applications for fecal immune and inflammatory biomarkers within ruminant veterinary medicine. Infectious enteritis in ruminants can be caused by a range of viral, bacterial or eukaryotic pathogens (Chigerwe and Heller 2018), and improving the monitoring, prevention, and treatment of disease could improve welfare, and prevent economic losses. Additionally, resistance to anthelminthics, the primary treatment option for gastrointestinal helminth infections, is becoming a growing global concern, necessitating improved monitoring and treatment regimes. Targeted selective treatment (TST; where only the most impacted individuals in a herd are selected for treatment) is an alternative to traditional, blanket anthelminthic treatment programs (Charlier et al. 2014; van Wyk et al. 2006). TST requires comprehensive metrics for animal health, of which fecal antibodies and inflammatory biomarkers are strong candidates for inclusion (Cooke et al. 2019). Similarly, for prevention of coccidiosis, timing of treatments is critical and should be coordinated with the parasitic life cycle (Bangoura and Daugschies 2019). Certain anticoccidials are only effective during the intracellular stages of the protozoans' life‐cycle, and drug administration should ideally be metaphylactic (before clinical signs appear) (Enemark, Dahl, and Enemark 2015; Greif 2000). It can be difficult to determine the state of the infection in animals in vivo, however, therefore treatment regimens often rely on historic knowledge of outbreaks on the farm. If fecal biomarkers were able to be correlated with specific parasitic life‐stages, this would provide an accurate way to determine the most effective treatment regime. Finally, Johne's disease progression is associated with a switch from a Th‐1 immune response to a Th‐2 response, and increased bacterial shedding (Magombedze, Eda, and Ganusov 2014). Current diagnostic tools for Johne's disease include serum or milk antibody tests, fecal culture or fecal PCR. Fecal antibody detection could be a possible avenue for cheap and noninvasive monitoring of disease progression, particularly for non‐milk producing animals, in addition to measuring treatment responsiveness.

Using the methods and antibodies developed for cattle, we were able to detect IgA, IgG, and lactoferrin in sheep and goat fecal samples, suggesting the same methods may be able to be used in a range of species within the Bovidae family. One wider application of these fecal biomarkers beyond veterinary diagnostics would be to study animal health in free‐living wildlife populations. Identifying overt clinical symptoms of disease in wildlife is extremely challenging (Ryser‐Degiorgis 2013); invasive sampling is often not possible, and non‐invasive ways to track changes in condition (such as body condition scoring) can be difficult to do accurately, particularly over the longitudinal timeframes needed to identify deteriorations in health. Ecoimmunology (the study of immunological variation within ecological settings) is at the forefront of the development of new noninvasive ways to monitor wildlife health, for example, the use of fecal antibodies to identify trade‐offs between reproduction and parasite resistance in wild Soay sheep (Hayward et al. 2019). The predictive power of many simple proxies of immune state is still limited (Downie et al. 2024), however, these results suggest that using fecal lactoferrin as a marker of gastrointestinal inflammation could provide novel insights into wild bovids' responses to infectious challenges. This is particularly important for infections that are considered subclinical and are therefore difficult to monitor in situ. Additionally, fecal biomarkers provide the ability to monitor wild animal health and immune states longitudinally, enabling a greater understanding of gut responses to disease, parasites, and environmental changes. We found no variation in mean lactoferrin concentrations between species, in contrast to previous work that found sheep and deer were significantly lower than cattle (Cooke et al. 2020). Fecal lactoferrin could therefore be a useful marker of gut health in multi‐host studies, particularly where co‐infections or unknown parasites may be present, thus limiting the use of parasite‐specific antibodies.

## 5 Conclusions

Building on previous work using fecal IgA, IgG, and lactoferrin in cattle, we suggest fecal lactoferrin is a useful biomarker of gut inflammation in response to gastrointestinal infections. Further validations are still needed before it can be used as a diagnostic tool, including with larger sample sizes and a wider range of infectious agents and disease severity, however these initial findings are promising. Longitudinal sampling of disease progression (and resolution), for example, is needed to determine its sensitivity and specificity. Fecal IgA was also significantly higher in cattle with infectious gastrointestinal pathologies than controls, suggesting fecal antibody detection could be a useful tool in monitoring gastrointestinal functionality. It did not significantly differ between infectious pathologies and asymptomatic Johne's disease, or noninfectious pathologies, which was predicted due to the multiple nonspecific functions of sIgA against a range of infectious and noninfectious pathological agents. Fecal IgG was not significantly different between groups, although the lowest concentrations were found in the controls. Since IgG is closely linked to the recognition of—and immune defense against—pathogenic gastrointestinal helminths, including more animals with these pathologies might reveal clearer differences in IgG between infections and control groups. Similarly in sheep, nonsignificant positive associations were seen between FEC and all biomarkers; including a larger sample size, with a wider range of FECs might make these relationships clearer.

Overall, these assays provide an easy, cost‐effective and noninvasive method to monitor animal health and immune responses to infectious or ecological challenges. They could increase the capabilities of livestock owners and vets to monitor gastrointestinal disease progression, and inform treatment regimes, through the ease of fecal sample collection. Furthermore, studies of parasite infections in wild or free‐living populations often focus on pathogen presence or burden, without the ability to quantify the physiological impact these are having on the host. Incorporating immune and inflammatory biomarkers into already existing frameworks of functional marginality (e.g., Shultz, Britnell, and Harvey 2021) or as proxies of immune phenotypes (Downie et al. 2024) will increase our understanding of the fitness consequences of infections at individual and population levels.

## Conflicts of Interest

The authors declare no conflicts of interest.

## Supporting information

Supporting information.

## Data Availability

The data that support the findings of this study are available from the corresponding author upon reasonable request.
