# Supplementary material for: Antibodies and Inflammation: Fecal Biomarkers of Gut Health in Domestic Ruminants
Source: J Exp Zool A Ecol Integr Physiol. 2025 Jan 22;343(4):468–79. doi: 10.1002/jez.2896 (PMC11959687; doi:10.1002/jez.2896)
Supplement: Supplementary file 1 — Supporting information. [file JEZ-343-468-s001.docx]

Supplementary Materials 1 – Equations Used

$$Linearity \left( \% \right)=\left( {C_{1}}/{({C_{2}}/{df}} \right))*100$$

***Equation 1:*** *Calculation of linearity from serially diluted samples.* C_1_= starting concentration, C_2_=diluted concentration, df= dilution factor used (1.5 for IgA, 2 for IgG and Lactoferrin).

$$Recovery \left( \% \right)=\left( {(C_{s}-C_{u}}/S \right))*100$$

***Equation 2:*** *Calculation of recovery from spiked samples.* C_s_= concentration of spiked sample, Cu=concentration of un-spiked sample, S= spike concentration*.*

$$LoB={Mean}_{Blanks}+1.645({Standard Deviation}_{Blanks})$$

***Equation 3:*** *Limit of Blanks (LoB).* Using optical densities at 450nm.

$$LoD=LoB+1.645({Standard Deviation}_{Lowest sample concentration})$$

***Equation 4:*** *Limit of Detection (LoD).* The low sample concentration used was the lowest dilution of the reference material (standard) for each assay. The LoD was then converted into an estimated lactoferrin concentration using a four - parameter logistic curve (Cooke et al., 2020).

$$Concentration \left( \frac{ng}{g} \right)=\frac{Concentration \left( \mu g \right)*extract volume \left( \mu l \right)*dilution factor*1000}{faecal weight \left( g \right)*sample volume \left( \mu l \right)}$$

***Equation 5:*** *Conversion of concentration from µg/ml to ng/g faecal matter.* Extract volume = volume of solvent used plus faecal weight (mg). This ensures an accurate estimation of the final concentration, as the feacal extraction is actually a suspension of the aqueous and solid (faecal) portions (Palme et al., 2013).

Supplementary Materials 2 – Inter-Specific Variation

Faecal IgA, IgG and lactoferrin were successfully measured in sheep and goat samples using a method developed for cattle. Concentrations were variable for all species. Sheep and goat concentrations were within the range of cows, however the minimum concentration for sheep was lower than for cattle across all assays. Species were not significantly different for IgG or lactoferrin, but sheep had significantly lower IgA than cattle (*p*=0.04, F_2,26_=1.07).


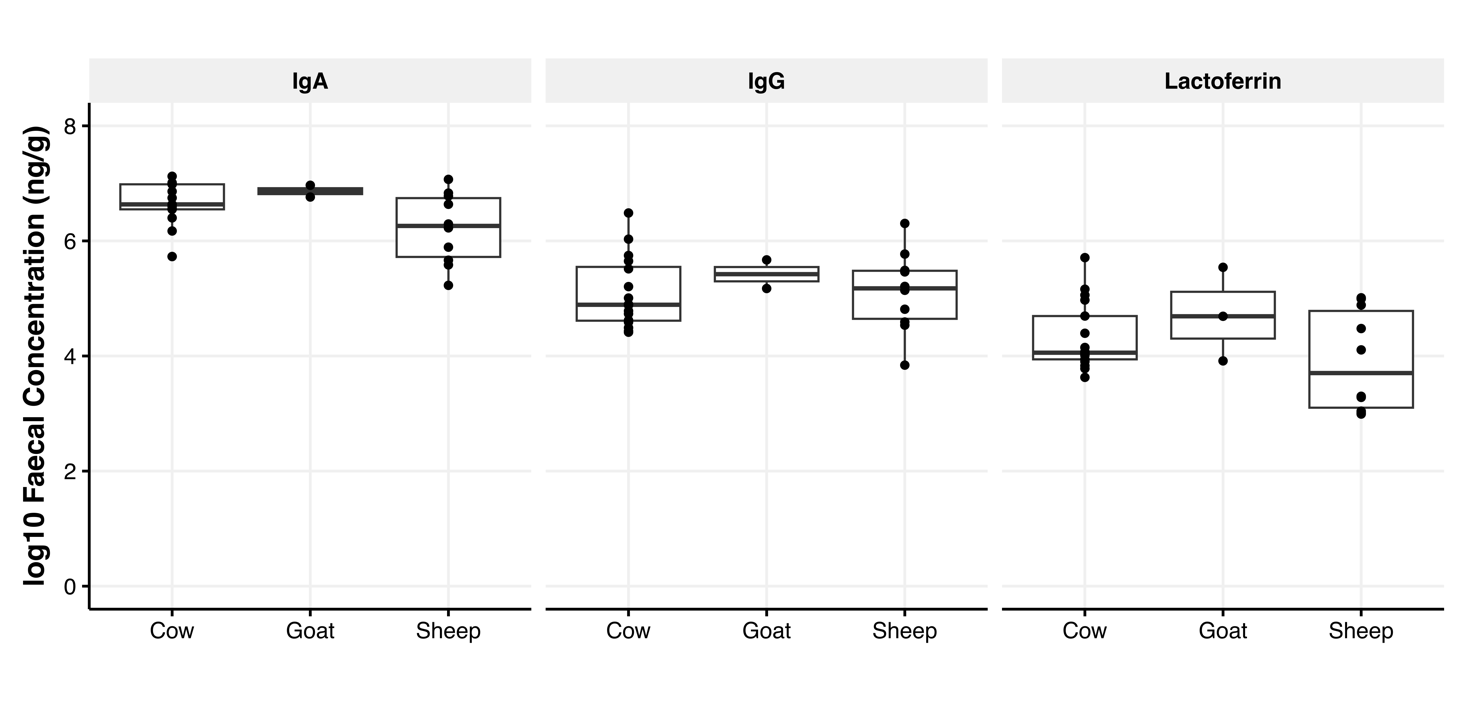
**Figure S1: Faecal IgA, IgG and lactoferrin was successfully detected in sheep, goat, and cow samples, with variable concentrations (log10 ng/g).**
